# Trauma team activation and triage of severely injured patients at one non-trauma-center hospital in Stockholm

**DOI:** 10.1186/s13049-024-01295-x

**Published:** 2024-11-27

**Authors:** Oscar Lapidus, Denise Bäckström, Folke Hammarqvist, Andreas Wladis, Rebecka Rubenson Wahlin

**Affiliations:** 1 Department of Clinical Science, Intervention and Technology, Karolinska Institutet, Stockholm, Sweden; 2 Department of Biomedical and Clinical Sciences, Linköping University, Linköping, Sweden

**Keywords:** Trauma, Triage, Trauma team activation, The Swedish Trauma Registry, Undertriage, Non-trauma-center hospital

## Abstract

**Background:**

In 2017 the Swedish public insurance company Löf published national guidelines for in-hospital trauma team activation (TTA), which are now widely adopted in Sweden. No studies have examined triage accuracy at non-trauma-center hospitals in the Stockholm trauma system since the implementation of the new TTA criteria.

**Aim:**

To assess trauma triage accuracy at one non-trauma-center hospital in Stockholm.

**Methods:**

3528 trauma patients treated at Södersjukhuset during 2019–2022 were acquired from the Swedish Trauma Registry (SweTrau) to calculate TTA triage accuracy. Undertriage was defined in accordance with national guidelines as patients with a New Injury Severity Score > 15 who did not prompt level 1 TTA on arrival to hospital.

**Results:**

In total there were 849 severely injured patients during the study period, of which 2.2% (*n* = 19) prompted TTA level 1, corresponding to an undertriage of 98% (*n* = 830). Of the 849 severely injured patients, 41% (*n* = 348) prompted TTA level 2 whereas the remaining 57% (*n* = 482) prompted no TTA on arrival to hospital. There were a total of 3046 patients prompting TTA during the study period, but only 19% (*n* = 19) of level 1 and 12% (*n* = 348) of level 2 patients were severely injured, and 45% had a NISS ≤ 3.

**Conclusion:**

Undertriage of severely injured trauma patients was 98% according to the definition specified by Swedish trauma triage guidelines, higher than reasonably acceptable. There is considerable overtriage with non-severely injured patients prompting TTA. However, the suitability of using NISS > 15 to retrospectively define the need for TTA is debatable as this does not always correlate with the fulfillment of the TTA criteria. Further investigation of adherence to trauma triage guidelines in clinical practice may be of value to improve triage accuracy in organized regional trauma systems.

## Background

To decrease mortality and morbidity associated with severe traumatic injuries, trauma systems worldwide have adapted triage tools to rapidly identify patients in need of urgent medical attention and in-hospital trauma team activation (TTA) [1–5]. Trauma team constellations may vary between institutions but usually consist of a multidisciplinary team of healthcare professionals capable of providing resuscitation and rapid assessment of patients suffering a broad spectrum of traumatic injuries [6]. Implementation of specific trauma center hospitals has also become more prevalent and has been shown to increase survival for the most severely injured patients [7].

In 2017 the Swedish insurance company Löf and a multidisciplinary group of experts from relevant medical specialty associations published national guidelines for TTA as the result of an extensive evaluation of the existing evidence for in-hospital TTA [8]. The criteria have since been widely implemented at emergency hospitals in Sweden. These guidelines advocate a two-tiered TTA system, and specify a set of physiological and anatomical criteria which, if fulfilled, should prompt level 1 TTA; there are also a set of criteria based on the mechanism of injury which should prompt level 2 TTA [9]. The guidelines also state that patients with a New Injury Severity Score (NISS) > 15 who do not prompt level 1 TTA are to be considered undertriaged, and declare this should be used as a quality indicator when reviewing the provided trauma care [9]. The intended use of the TTA criteria according to the guidelines is for emergency medical services (EMS) to pre-alert the hospital before arrival, and for triage staff in the emergency department to decide the appropriate in-hospital response based on TTA criteria, choosing either TTA (level 1 or 2), or deciding against prompting TTA [9].

According to a prehospital transport directive for trauma patients in Stockholm, all patients with obvious severe injuries should be transported to the regional trauma center (Karolinska University Hospital) by EMS instead of the nearest hospital [10]. These prehospital criteria are almost identical to those for in-hospital level 1 TTA, which theoretically implies no level 1 TTA patients are supposed to be treated at non-trauma-center hospitals if EMS perfectly adhere to the prehospital directive. Consequently, the number of level 1 TTA at non-trauma-centers is expected to be very low as these patients are ideally transported to the regional trauma center. However, each year several severely injured trauma patients are still admitted to the emergency departments at various non-trauma-center hospitals in Stockholm [11]. The full extent to which this occurs is not currently known, and no recent studies have examined triage accuracy at non-trauma-center hospitals in Stockholm.

### Aim

To assess trauma triage accuracy at one non-trauma-center hospital in Stockholm.

## Methods

### Study design

The present study was a retrospective single-center cohort study examining patients presenting to the emergency department at Södersjukhuset in Stockholm, Sweden. The study was reported in accordance with the Strengthening the Reporting of Observational studies in Epidemiology (STROBE) guidelines [12].

### Ethical considerations

This study was approved by the Ethical Review Authority in Sweden (2022-06727-01).

### Setting

The present study was performed in the Stockholm region (swe: Region Stockholm) with a population of 2.4 million inhabitants in 2022, distributed over 6514 km^2^ [13]. The Stockholm metropolitan area is served by a single trauma center hospital as well as four centrally located non-trauma-center emergency hospitals, with two additional emergency hospitals in the north and south of the region (Norrtälje and Södertälje). The present study examined data from Södersjukhuset, one of the centrally located non-trauma-center emergency hospitals and the largest by number of acute patients. In 2022 Södersjukhuset accounted for 29% of the total number of patients with NISS > 15 reported to SweTrau in the Stockholm trauma system, but in addition to primary transports this also includes secondary transfers of patients between hospitals [14].

### Participants

The study cohort included all trauma patients at Södersjukhuset from 2019 to 2022 registered in The Swedish Trauma Registry (SweTrau). Study inclusion criteria were age ≥ 15 years and either NISS > 15 or TTA on arrival to the emergency department, the latter two also being inclusion criteria for SweTrau [15]. Secondary transports between hospitals were excluded. In total there were 3528 eligible trauma patients. A subgroup analysis of severely injured geriatric patients (age ≥ 65 years & NISS > 15) was also performed as undertriage may be especially prevalent in this subgroup [16]. Because the triage definition specified in the national guidelines (NISS > 15) may be considered somewhat arbitrarily, a subgroup analysis of patients with NISS ≥ 25 was also performed to include a more strict definition of patients in need of level 1 TTA.

### Variables

Study outcome was the rate of undertriage of severely injured patients (NISS > 15). Undertriage was defined in accordance with Swedish national guidelines as NISS > 15 patients who did not prompt level 1 TTA on arrival to the emergency department [9]. Triage accuracy was determined for each patient using the variables for TTA status ‘ed_tta’ and ‘TraumaAlarmAtHospital’, and injury severity using the ‘NISS’ variable. Primary transports were identified using the ‘pre_transport’, ‘hosp_transferred’ and ‘TraumaAlarmCriteria’ variables coded as 999 (not applicable) in the SweTrau data.

### Data sources

The Swedish Trauma Registry (SweTrau) is a national quality registry which aims to monitor and improve trauma care by collecting and providing data that can be used for research and to identify areas for improvement in clinical trauma care [15]. Inclusion criteria for SweTrau are TTA (level 1 or level 2) regardless of injury severity, as well as all trauma patients who are retrospectively found to have a NISS > 15.

### Bias

By design, the present study is subject to selection bias towards trauma patients with less obvious injuries, but this intended as the purpose of the study was to explore trauma triage accuracy at non-trauma-center hospitals. However, the study may also be subject to a registration bias inherent to SweTrau registry as the inclusion criteria are either TTA on arrival or NISS > 15 regardless of TTA, because patients prompting TTA are easy to identify by reviewing TTA records, but patients fulfilling only the NISS criteria may be more difficult as it requires review of all medical records of patients presenting to the emergency department following a traumatic mechanism of injury. However, any non-TTA patients with NISS > 15 who were not registered in SweTrau affects both the numerator and the denominator in the equation for triage accuracy. Consequently, the measure of undertriage is a theoretical ‘minimum’ as there could be more unregistered undertriaged patients but likely no more unregistered TTAs.

### Study size

The present study used all eligible patients during the study period of 2019–2022. Including patients before 2019 the study period was deemed unsuitable because of lacking SweTrau coverage at Södersjukhuset.

### Statistics

Descriptive Statistical analysis was performed with a significance level of 0.05. The Shapiro-Wilk tests were used to determine which variables had normal distribution. Median values and quartiles, as well as percentages and the total number of patients was reported for descriptive statistics. The Chi-square test was used to statistically compare undertriage between younger and older patients for the subgroup analysis.

## Results

The cohort was comprised of 58% (*n* = 2061) male and 42% (*n* = 1467) female patients, with a median (Q1, Q3) age of 59 (38, 77) years. Baseline characteristics of the study cohort stratified by NISS > 15 and NISS < 15 are presented in Table 1.

**Table 1:** Baseline characteristics of the trauma cohort at Södersjukhuset. Subdivided by severely injured patients (NISS > 15) and patients included only because of TTA on arrival to hospital (NISS < 15)

Demographics	NISS > 15 *n* = 849		NISS < 15 *n* = *2 679*	
**Patient characteristics**:				
Sex (male: female), % (n)	59:41	(497:352)	58:42	(1564:1115)
Age, median (Q1, Q3)	71	(56, 83)	55	(35, 75)
**Injury severity and mortality**:				
ISS, median (Q1, Q3)	17	(13, 20)	2	(1, 5)
NISS, median (Q1, Q3)	22	(17, 27)	2	(3, 8)
30-day mortality, % (n)	11%	(*n* = 92)	2.5%	(*n* = 67)
**Injury type**:				
Blunt, % (n)	98%	(835)	94%	(2516)
Penetrating, % (n)	1.6%	(14)	6.1%	(163)
**Injury intention**:				
Accident, % (n)	94%	(799)	92%	(2 453)
Violent assault, % (n)	5.3%	(45)	6.5%	(174)
Self-inflicted, % (n)	0.6%	(5)	1.9%	(52)
**Mechanism of injury**:				
Low fall	56%	(472)	39%	(1047)
High fall	20%	(171)	17%	(464)
Bicycle accident	11%	(94)	9.6%	(257)
Hit/struck by blunt object	5.4%	(46)	8.5%	(229)
Motorcycle accident	2.4%	(20)	2.9%	(77)
Injured pedestrian	1.8%	(15)	9.6%	(54)
Injured by knife/sharp object	1.6%	(14)	6.2%	(165)
Motor vehicle accident	1.4%	(12)	13%	(355)
Explosion injury	0%	(0)	0.2%	(6)
Gun shot wound	0%	(0)	0.1%	(3)
Other vehicle	0.5%	(4)	0.6%	(17)
Other mechanism of injury	0.1%	(1)	0.2%	(5)

**Fig. 1:**
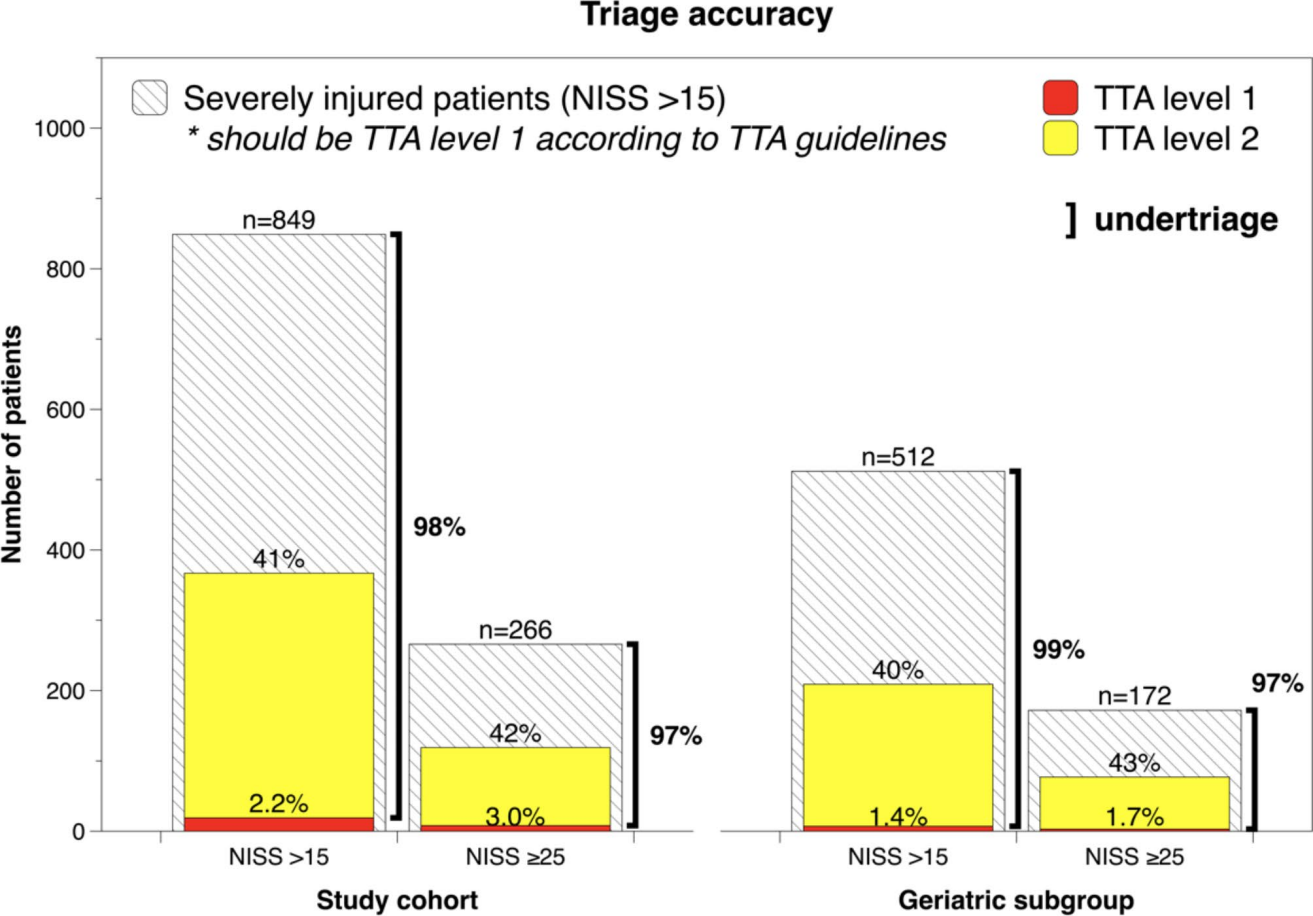
Triage accuracy for severely injured patients at Södersjukhuset

In total, there were 849 severely injured patients (NISS > 15) during the study period, for whom the rates of TTA level 1 and level 2 were 2.2% (*n* = 19) and 41% (*n* = 348); the remaining 482 patients (57% of cases) prompted no TTA on arrival (Fig. 1).

The rate of undertriage of severely injured patients, defined as the proportion of patients with NISS > 15 who did not prompt TTA level 1, was 98% (*n* = 830) (Fig. 1). The most frequent mechanism of injury for severely injured patients was a low fall constituting 56% of cases (*n* = 472), followed by high fall at 20% (*n* = 171) and bicycle accidents at 11% (*n* = 94) (Table 1). Of 849 severely injured patients, 266 had NISS ≥ 25. In this group, 3.0% (*n* = 8) and 42% (*n* = 111) triggered a TTA level 1 and 2 respectively, whereas 55% (*n* = 147) prompted no TTA on arrival to the emergency department, corresponding to an undertriage rate of 97% for the most severely injured patients.

A total of 3046 patients triggered a TTA at Södersjukhuset during the study period, of which 3.3% (*n* = 101) were TTA level 1 and 97% (*n* = 2945) were level 2 (Table 2).

**Table 2:** Number of TTAs and severely injured patients per year at Södersjukhuset

Year	TTA level 1 % of TTA (*n*)		TTA level 2 % of TTA (*n*)		NISS > 15, no TTA *n*
2019	4.2%	(30)	96%	(668)	114
2020	3.6%	(23)	96%	(610)	121
2021	2.7%	(22)	97%	(784)	117
2022	2.9%	(26)	97%	(863)	130
**Total**	**3.3%**	**(101)**	**97%**	**(2945)**	**482**

**Fig. 2:**
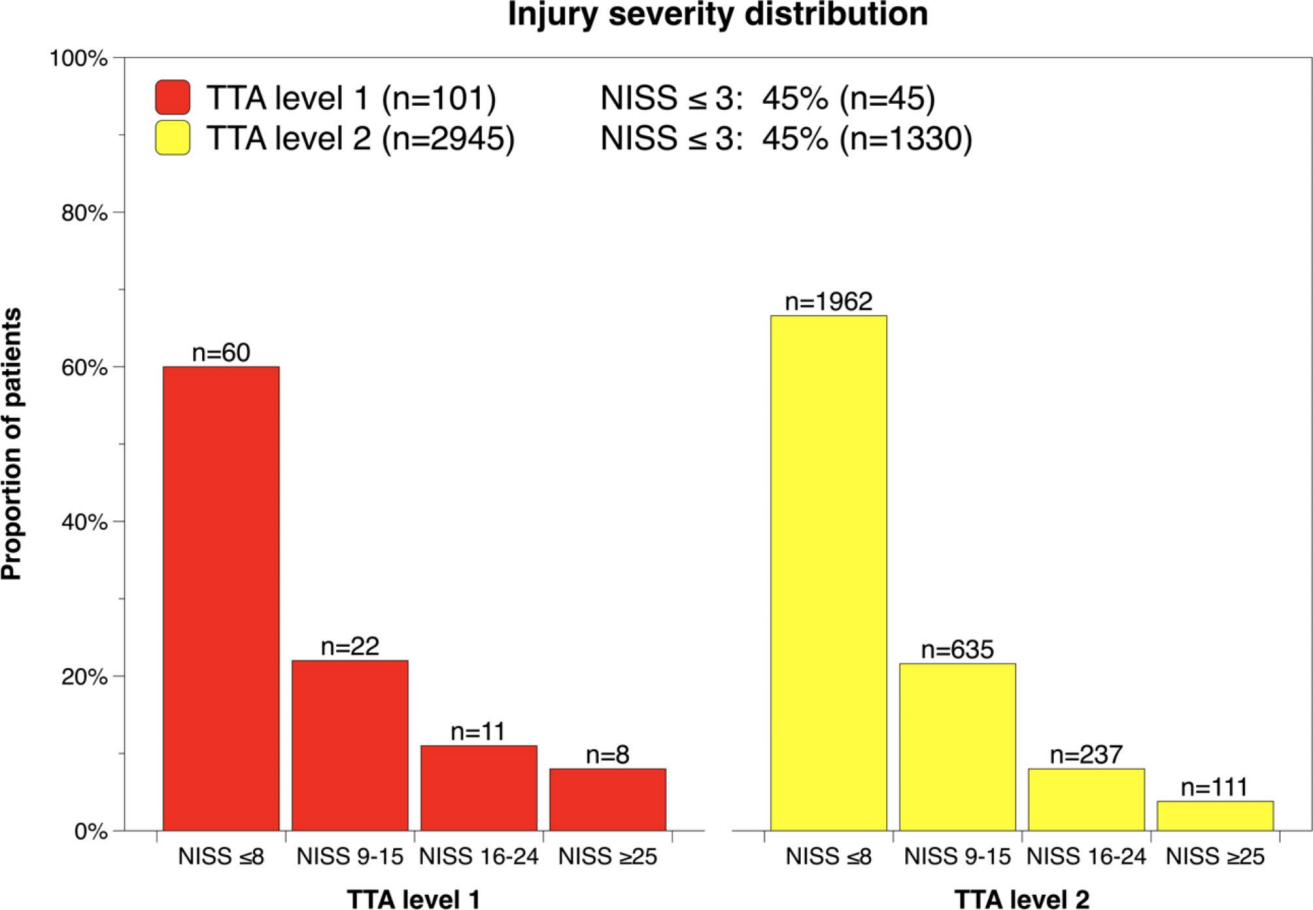
Injury severity distribution for patients prompting TTA

The majority of patients prompting TTA were not severely injured, as only 19% (*n* = 19) of level 1 and 12% (*n* = 348) of level 2 patients had a NISS > 15 (Fig. 2). Furthermore, 45% (*n* = 45) of patients prompting level 1 TTA and 45% (*n* = 1330) of patients prompting level 2 TTA had a NISS score less than or equal to 3 (Fig. 2).

There were 512 severely injured geriatric patients (age ≥ 65 years and NISS > 15), constituting approximately 60% of severely injured patients in the examined cohort. Of these geriatric patients, 1.4% (*n* = 6) triggered TTA level 1, 40% (*n* = 202) TTA level 2 and 59% (*n* = 303) no TTA, corresponding to an undertriage of 99% (*n* = 437) (Fig. 1). Severely injured geriatric patients (age ≥ 65 years & NISS > 15) had significantly higher undertriage at 99% compared to 96% for patients aged 15–64 years (*p* = 0.034).

## Discussion

The present study, which aimed to assess trauma triage accuracy at one non-trauma-center hospital, found that undertriage of severely injured trauma patients was 98%. Furthermore, the majority of TTA patients were not severely injured as only 19% of patients prompting level 1 TTA and 12% of level 2 TTA had a NISS > 15, hence triage accuracy was poor as there were high rates of both undertriage as well as overtriage. The rate of undertriage was far in excess of the generally accepted limit of no more than 5% undertriage recommended by the American College of Surgeons (ACS) [17]. However, the definition of undertriage used by ACS differs slightly from that of the present study as it uses the total number of patients who did not prompt the highest level of TTA as the denominator in the equation for triage accuracy, whereas the Swedish guidelines instead use the total number of patients with NISS > 15 [9, 17]. There were no recent studies from the Stockholm trauma system available for direct comparison of results, but Granström et al. found an undertriage of 7–10% in their study examining triage accuracy at the regional trauma center hospital in Stockholm during 2011–2013, although the definition of undertriage differed slightly from that of the present study as they used the Injury Severity Score (ISS) to define the need for TTA, whereas the present study used the NISS [18]. Similarly, in the validation study for the national guidelines, Linder et al. found an undertriage of 5–7% using the new TTA criteria [19]. However, although a well-made study, the authors used the ACS definition of undertriage, which may arguably not be the most appropriate method for calculating triage accuracy, as highlighted by Peng and Xiang [19, 20]. However, Linder et al. also calculated the number of patients with ISS ≥ 15 who did not prompt level 1 TTA according to the Peng method, resulting in an undertriage of 54% and 49% with the old and new TTA criteria respectively (*p* = 0.565), which is closer to the 98% observed in the present study; however, the cohorts differ in terms of patient demographics and the prehospital setting of the regional trauma systems.

The cause of the poor triage accuracy observed in the present study is not known, but it is possible that the TTA criteria are not used when deciding the need for TTA, despite the stated clinical routine at Södersjukhuset being to assess whether any of the specified TTA criteria are fulfilled when deciding the need for in-hospital TTA. Such a deviation may be due to lacking knowledge of the TTA criteria but could also be because clinicians performing triage decide not to follow the national guidelines. Instead, some alternative method for evaluating the need for TTA may be chosen, such as simply adhering to whichever level of TTA is suggested by EMS without reevaluating if any TTA criteria are fulfilled, which could also explain the high rate of overtriage (non-severely injured patients triggering TTA) seen in the present study. However, this method would not be in accordance with the local clinical routine or the national guidelines as they specifically state that the intended use is for EMS to pre-alert the hospital, after which triage staff in the emergency department decide the appropriate in-hospital response based on the fulfillment of the TTA criteria [9]. However, as the present study was not designed to assess adherence to TTA criteria, whether or not triage was performed in accordance with the national trauma triage guidelines and the TTA criteria remains unknown but warrants further investigation. Another possible explanation for the high rate of undertriage may be a reluctance in making the decision to prompt the level 1 TTA (full multidisciplinary trauma team), instead choosing a level 2 TTA as this generates a more limited in-hospital response.

While adherence to the trauma triage guidelines may be poor in clinical practice, it is also possible that the TTA criteria are inherently unable to accurately identify severely injured patients in the local trauma cohort at Södersjukhuset. Although the TTA criteria were validated in a recent high-quality multicenter study, the setting of the examined validation hospitals differed substantially from that of the present study which was performed in a regional trauma system with a strict prehospital selection of patients who are suspected to be severely injured to the regional trauma center hospital in accordance with a prehospital directive in Region Stockholm [21]. The national guidelines also heavily emphasize high-energy mechanisms of injury in addition to specific physiological and anatomical criteria, most of which are the result of high-energy mechanisms; for example, a fall from 5 m or higher should generate a level 2 TTA according to the national guidelines [9]. In the present study the most common mechanism of injury for severely injured patients was a low fall, defined in SweTrau as less than 1.5 × patient height, constituting 56% of cases, which is not a mechanism of injury that should prompt TTA according to the guidelines [9]. Although the second most common mechanism of injury was a high fall at 20%, most of the severely injured patients prompting level 2 TTA likely did not meet any of the level 2 TTA criteria as these are based only on the mechanism of injury; however, assessing the fulfillment of TTA criteria was beyond the scope of the present study.

In the present study, the median age was 59 years for all included patients and 71 years for patients with NISS > 15; in contrast, the cohort in the validation study had an average age of 36–40 years [19]. Although the sensitivity of the TTA criteria may have been sufficient in that cohort, the national guidelines do not consider that elderly patients require less force to sustain severe injuries, and as previously mentioned the mechanisms of injury may not be the same as for younger patients. Preexisting conditions and cognitive impairment may also interfere with triage decisions when there are other medical concerns or if there is uncertainty regarding whether the patient has suffered trauma in the first place, further complicating the question of how to ensure an appropriate level of care for these patients [22, 23]. Furthermore, the prehospital directive for trauma patients in Region Stockholm is very similar to the level 1 TTA criteria, and strict adherence to this transport directive should ideally result in no patients fulfilling level 1 TTA criteria being transported to non-trauma-center hospitals as they also meet the criteria of the trauma directive and are transported to the regional trauma center by EMS. Consequently, the TTA criteria may arguably not be suitable to be used at non-trauma-center hospitals in a regional trauma system subject to prehospital selection of major trauma patients away from these hospitals; however, there is also a lack of suitable alternatives.

As previously mentioned, the present study found that a high proportion of patients in the severely injured cohort were geriatric, defined in this study as ≥ 65 years old. Whether or not these patients should be classified as geriatric based only on their chronological age is debatable, but one may argue that the fact that most of these patients sustained severe injuries from a low-energy mechanism of injury implies an inherent vulnerability, which would justify classifying them as geriatric patients [24]. Furthermore, several previous studies have shown that age alone is an independent risk factor for mortality and morbidity in trauma, hence it was considered suitable to be used to define the geriatric subgroup in the present study; there was also a lack of suitable data regarding preexisting conditions and comorbidities in SweTrau [25–27].

The finding that the most prevalent mechanism of injury in the severely injured trauma cohort was a low fall is also interesting in relation to the triage definition used in the present study, as one may argue that low-energy mechanisms rarely cause time-critical injuries, and that these patients do not strictly require level 1 TTA regardless of scoring above a certain (often arbitrary) threshold in terms of NISS or some other coring system [28]. Which patients actually require TTA and which do not is debatable and there is a lack of high-level evidence, but according to the Swedish national guidelines the purpose of TTAs is to ensure the highest quality of care for the most severely injured patients [9]. However, in the present study the severe mismatch in triage resulted in the most severely injured patients initially receiving the lowest level of care, while at the same time there were many TTAs for patients who were not severely injured. Previous investigations have also concluded that geriatric trauma patients may sustain severe multisystem injuries from low-energy mechanisms, and that ground-level falls are often misclassified as not serious by both healthcare professionals and current trauma triage protocols [22, 23, 29].

Whether or not we should consider the de-facto true requirement for TTA to be the post-hoc injury severity or the fulfillment of specified TTA criteria itself is debatable, and the evidence regarding which patients require TTA and which do not is limited. However, some post-hoc definition of injury severity (ex. ISS or NISS) is often used to determine the need for TTA when retrospectively assessing trauma triage accuracy, and one of the most frequently used definition in existing literature is NISS > 15 [30]. However, the authors acknowledge this may arguably not be a perfect classifier of injury severity and there are multiple other methods which could have been used; however, the NISS > 15 definition is the only method suggested in national guidelines and allows for uncomplicated comparison with the literature on this topic [9, 31]. Previous authors have also highlighted various methods for calculating undertriage, but the method described by Peng and Xiang was chosen for the present study as this is the preferred method according to Swedish trauma triage guidelines [9, 20]. This method is a post-hoc definition based on injury severity (calculated as NISS) [32]. Consequently, the present study made no effort to assess whether the specified TTA criteria were fulfilled or not as the undertriage definition was based only on NISS. However, clinical adherence to triage guidelines in the Stockholm trauma system is also an important measure of triage accuracy and warrants further investigation.

## Conclusion

Undertriage of severely injured trauma patients was 98% according to the definition specified by Swedish trauma triage guidelines, higher than reasonably acceptable. There is considerable overtriage with non-severely injured patients prompting TTA. However, the suitability of using NISS > 15 to retrospectively define the need for TTA is debatable as this does not always correlate with the fulfillment of the TTA criteria. Further investigation of adherence to trauma triage guidelines in clinical practice may be of value to improve triage accuracy in organized regional trauma systems.

## Data Availability

The data will not be published but is available from the corresponding author.
